# Relationship Between Physical Activity, Parental Psychological Control, Basic Psychological Needs, Anxiety, and Mental Health in Chinese Engineering College Students During the COVID-19 Pandemic

**DOI:** 10.3389/fpsyg.2022.802477

**Published:** 2022-03-08

**Authors:** Zongyu Liu, Meiran Li, Chuanqi Ren, Guangyu Zhu, Xiuhan Zhao

**Affiliations:** School of Physical Education, Shandong University, Jinan, China

**Keywords:** physical activity, parental psychological control, basic psychological needs, anxiety, mental health

## Abstract

The issue of mental health among college students is of increasing concern during the COVID-19 outbreak. Since course characteristics of engineering college students determine the particularities of their mental health, the specific objectives of this study were: (1) to analyze the relationship between physical activity, parental psychological control, basic psychological needs, anxiety, and mental health in Chinese engineering college students during COVID-19 pandemic; and (2) to examine the mediation effect of anxiety between the relationship of basic psychological needs and mental health. A cross-sectional study was conducted among several universities in Shandong Province, China. We randomly selected 254 Chinese engineering college students from these colleges. Participants who were given questionnaires completed the Physical Activity Rating Scale (PARS-3), Basic Needs Satisfaction in General Scale (BNSG-S), Parental psychological control Questionnaire, the Beck anxiety inventory (BAI), and the Kessler 10 (K10) scale. The mediation model was conducted to assess the mediation effect of anxiety between the relationship of basic psychological needs and mental health. Among 254 Chinese college students majoring in engineering, the results showed that their mental health was in the mid-level range. Besides, physical activity and basic psychological needs is positively correlated with mental health, respectively, while parental psychological control is not correlated with mental health. Anxiety is negatively associated with mental health. Mediation analysis revealed that anxiety played a mediation role in the relationship between basic psychological needs and mental health. In conclusion, mental health of Chinese engineering college students deserves extensive attention during the COVID-19 pandemic. Proper intervention on physical activity, basic psychological needs, and anxiety may be beneficial to improve their mental health. In addition, meeting basic psychological needs is beneficial to reduce anxiety and improve mental health further.

## Introduction

The outbreak of COVID-19 represents a public health emergency of international concern, and citizens were urged to stay at home for quarantine measures that obligate individuals to stay home or significantly limit their out-of-home activity, which may have a negative psychological impact on them (Wathelet et al., 2020). In recent years, the mental health of college students has been a subject of social attention, especially with suicides among college students being reported from time to time (Chen et al., 2020). Universities closed in most of the regions as countries started implementing preventive quarantine and lockdown interventions, which may have an adverse implication on the mental health of college students. A web-based survey among 746,217 college students during the COVID-19 outbreak in China has found widespread mental health problems such as acute stress and depressive symptoms exist in college students (Ma et al., 2020). A recent study found that engineering students were twice as likely to experience mood and anxiety disorders compared with the general college population (Danowitz and Beddoes, 2018).

As a special group of students, engineering students’ learning levels are notoriously stressful and competitive, requiring long hours of study, training and practice, which ultimately affects their physical and mental health (Siddiqui et al., 2020). Compared with other professional disciplines (such as liberal arts and business), engineering students need to learn a large amount of knowledge in a limited time and may feel more psychological pressure due to their academic burden, fear of employment after graduation, and lack of confidence (Karthikeson and Jagannathan, 2016; Jensen and Cross, 2021). Therefore, it is of great significance to clarify the influencing factors of engineering college students’ mental health during the COVID-19 pandemic to formulate corresponding intervention measures and improve their mental health. The identification of influencing factors (such as physical activity) will help to formulate corresponding strategies to improve the well-being of engineering students and maintain their mental health under the condition of high intensity of study.

Physical activity is recognized as an important dimension that affects mental health. Studies have proven that regular physical activity significantly enhances the mental health and well-being of college students (Herbert et al., 2020). Regular physical activity protects college students from mental health problems and has a positive impact on mental health (Alhaqbani et al., 2020). Herbert et al. found that short-term exercise interventions may have beneficial effects on the mental health and well-being of college students (World Health Organization, 2010; Herbert et al., 2020). According to WHO, moderate-to-vigorous physical activity including daily activities that require energy expenditure can have a positive impact on the mental health of students (Tudor-Locke et al., 2001; WHO, 2020). For example, Xiang et al. (2020) also proved that college students engaged in high-level physical activity are less likely to have anxiety or depression than those engaged in low-level physical activity. Nonetheless, college students have been shown to spend less time on moderate and vigorous physical activity during COVID-19 confinement and require due attention (Rodriguez-Larrad et al., 2021).

Parental psychological control refers to parents’ attempts to interfere with their children’s mental and emotional development (Zhou et al., 2021). In China, parental control has been determined to be negatively correlated with children’s mental health, which will destroy children’s sense of autonomy at the appropriate age, thus leading to the misbehavior of teenagers and damaging their mental health (Zhou et al., 2017; Shek et al., 2019). Parental control includes behavioral control and psychological control (Barber, 1996). Studies have proved that parental behavioral control can promote the good development of children and adolescents, while psychological control is not conducive to their physical and mental health, and may inhibit their emotions (Barber et al., 2005; Wang et al., 2007; Sheldon, 2011; Zhou et al., 2021). However, one study demonstrated that parental control was positively associated with children’s mental health in China when used occasionally (Shek and Lee, 2007). The researchers argue that parental control can undermine a child’s sense of proper age autonomy, leading to adolescent misbehavior and mental health damage (Barber and Harmon, 2002; Shek et al., 2018).

Basic psychological needs theory was proposed by Deci and Ryan, who believed that the competence need, autonomy need and relatedness need are the basic nutrients for human growth, integrity, and health (Ryan and Deci, 2000). Self-determination theory is the core of the theory of basic psychological needs (Orkibi and Ronen, 2017). According to the self-determination theory, whether the basic psychological needs can be met not only affects the possibility of individual development, but also reflects the individual’s mental health (Shannon et al., 2019). When the psychological needs are continuously met, people will maintain healthy physical development, optimal functional state and a high degree of happiness (Junior et al., 2019). Research shows that positive functioning and optimal mental health follow when basic needs are met (Deci et al., 2001). Studies have proved that basic psychological needs are closely related to anxiety, depression and life satisfaction, and can enhance happiness and promote the healthy growth of individual mental health (Sheldon et al., 2001; Gunnell et al., 2013; Van den Broeck et al., 2016). Zhou et al. (2020) have revealed that anxiety, such as health anxiety or social anxiety, can adversely affect an individual’s physical and mental health.

**FIGURE 1:**
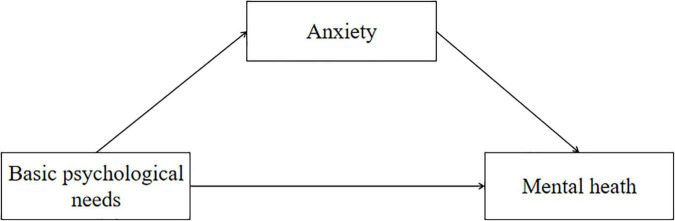
Structural framework.

There have been few studies on the mental health of engineering college students during the COVID-19 pandemic. Therefore, understanding the factors affecting the mental health of engineering college students is helpful to formulate corresponding intervention measures against the backdrop of the normalization of epidemic prevention and control in China. Based on this, the purpose of this study is to: (1) analyze the relationship between physical activity, parental psychological control, basic psychological needs, anxiety and mental health of Chinese engineering students during COVID-19; (2) To study the mediation role of anxiety in the relationship between basic psychological needs and mental health. The representation of the structural model is shown as follows (Figure 1).

## Materials and Methods

### Participants and Procedures

A total of 269 Chinese college students majoring in engineering in several universities in Jinan, Shandong province, were selected through convenience sampling in spring semester 2021. All investigators involved in the study received the required formal training. Before the research, researchers explain the concept and the purpose of this study to students who want to participate in this study. Participants were asked to fill out an electric questionnaire seriously if they have consented to the study. The trained researchers collected data on basic sociodemographic information, physical activity, parental psychological control, basic psychological needs, anxiety and mental health of college engineering students. The questionnaire was administered to students before their class and they were allocated 20 min for completion. After each interview, quality control was 100% checked on completed questionnaires. 254 valid questionnaires were obtained, with an effective rate of 95.2%. The Ethics Committee of Shandong University approved this study (No. 20190912).

### Instruments

All of the survey tools detailed below were used by the research team during school sessions to assess different variables. For the purpose of examining the differences by sex, we used age, height, weight, and whether or not a single parent lived in a household as basic demographic variables. Body mass index (BMI) was calculated as body mass in kilograms divided by stature in meters squared.

Physical activity assessment was performed using the physical Activity Rating Scale (PARS-3), modified by Liang and Liu (Liu et al., 2020), which measures intensity, duration, and frequency of physical activity. A 5-point Likert scale was used for quantification, with a score of 1–5 for each item. Total physical activity score = activity intensity score × (activity time score -1) × activity frequency score, the scoring interval is 0–100 points.

The Chinese version (Yu et al., 2012) of the General Scale of Satisfaction with Basic Needs (BNSG-S) (Gagné, 2003) was used to measure the satisfaction of basic psychological needs. BNSG-S consists of 21 items, including six items measuring competence needs, seven items measuring autonomy need, and eight items measuring relatedness needs. The items used a seven-point Likert scale ranging from 1 “not at all” to 7 “very much.” Across the scale, 12 items were rated positively, such as “I feel I can decide how to live my life,” and nine other items were rated negatively, such as “I often feel incompetent.” The higher the sub-scale score, the greater the satisfaction with corresponding needs.

Compiled by Wang et al. the Parental psychological control scale (Bleakley et al., 2016) consists of 18 items that measure parental behaviors such as feelings of guilt, withdrawal of love, and authoritarianism, such as “If I don’t see things my parents’ way, they will be less nice to me.” Teens were asked to report how well each program matched their parents’ actual situation. A five-point score is used, ranging from “completely inconsistent” to “very consistent,” and the higher the score is, the more serious the parental psychological control is.

Anxiety was assessed using the Beck Anxiety Inventory (BAI), which was designed by Aaron Beck in 1985 (Kim et al., 2020). The scale has a total of 21 items with a four-level score in each item. The main evaluation of anxiety is the extent to which participants are bothered by various anxiety symptoms. The rating level is divided into none; Mild, without much bother; Moderate, uncomfortable but tolerable; Severe, just barely tolerated. The score for this time is “none,” and so on, the more serious the score will be higher. The rough score is converted to the standard score using the formula y = int (1.19×). A higher score indicates more anxiety, while a lower score indicates less anxiety. BAI is helpful to understand an individual’s anxiety and the change of anxiety during treatment and is a clinical tool to analyze participants’ subjective anxiety symptoms.

The Kessler 10 Psychological Distress Scale, developed by Kessler and Mroczek (Kessler et al., 2002), is a short self-management rating Scale that can detect the risk of Psychological conditions in a crowd. The 10-item scale measured the frequency of non-specific mental health-related symptoms such as anxiety and stress levels experienced in the previous 4 weeks. Likert’s 5-point scoring method was used for each question, and 5∼1 points were scored all the time, most of the time, some of the time, occasionally and hardly. The higher the score, the worse mental health.

### Statistical Analysis

The original data were exported from the Wenjuanxing questionnaire platform.^1^ All of the statistical analyses were performed with SPSS version22.0 (IBM, Armonk, NY, United States) for windows. According to the research purpose, measurement data were expressed as the mean ± standard deviation (SD) and categorical variables were expressed as numbers (*n*) and percentages (%). Descriptive statistics were performed on all variables by chi-square test and *T*-test or analysis of variance (ANOVA) according to gender. Spearson’s correlation was used to examine the association between physical activity, parental psychological control, basic psychological needs, anxiety and mental health. Hayes’ PROCESS macro in SPSS (version 3.3) was performed in the mediation analysis (Hayes, 2013). The bootstrap method (sampling was repeated 5,000 times) was used to estimate 95% confidence intervals (CIs) for significance testing of mediating effects. When the CI did not include zero, the direct or indirect effect was considered significant. All variables were standardized before entering the mediation model.

## Results

A total of 254 Chinese engineering college students surveys were used in the analysis, including 241 (94.8%) males and 13 (5.1%) females. The mean (standard deviation) age of the participants was 18.93 (0.881) years. Among these samples, 67 (26.4%) of participants were from rural areas, and 187 (73.6%) were from urban areas. 22 (86.6%) lived in a single-parent family (Table 1). The mean (standard deviation) of physical activity, parental psychological control, basic psychological needs, anxiety and mental health was 32.53 (13.16), 2.61 (0.99), 3.05 (0.49), 32.53 (13.17), and 3.58 (90.85), respectively (Table 2). The results showed that only physical activity was significantly different between groups.

**TABLE 1:** General characteristics of participants according to gender among Chinese engineering college Students.

Variables		Total (*n* = 254)	Boys (*n* = 241)	Girls (*n* = 13)	T	*p*-Value
Age (years)		18.93 (0.881)	18.95 (0.889)	18.62 (0.650)	1.306	0.193
Place of residence, *n* (%)	Rural	67 (26.4)	64 (95.5)	3 (4.5)		
	Urban	187 (73.6)	177 (94.7)	10 (5.3)	-0.276	0.783
A single parent family or not (%)	Yes	22 (86.6)	22 (100)	0 (0)		
	No	232 (13.4)	219 (94.4)	13 (5.6)	-4.910	0.000***
Weight (Kg)		68.679 (11.027)	69.286 (10.871)	57.423 (7.449)	5.438	0.000***
Height (m)		1.771 (0.064)	1.778 (0.057)	1.637 (0.034)	3.882	0.000***
BMI (Kg/m^2^)		21.871 (3.122)	21.897 (3.161)	21.393 (2.313)	0.567	0.571
Overweight (%)	Yes	45 (17.7)	44 (18.3)	1 (7.7)		
	No	209 (82.3)	197 (81.7)	12 (92.3)	32.780	0.000***

Data were described as n (%) or mean ± SD. ***p < 0.001.

**TABLE 2:** Descriptive data of participants’ physical activity, parental psychological control, basic psychological needs, anxiety and mental health divided by gender.

	Total mean ± SD	Males’ mean ± SD	Females’ mean ± SD	T	*p*-Value
Physical activity	32.53 ± 13.16	25.14 ± 25.29	13.54 ± 12.59	2.235	0.001***
Parental psychological control	2.61 ± 0.99	2.60 ± 1.00	2.81 ± 0.87	−0.746	0.467
Basic psychological needs	3.05 ± 0.49	3.05 ± 0.49	2.97 ± 0.44	1.155	0.249
Anxiety	32.53 ± 13.17	32.34 ± 13.16	35.92 ± 13.41	−0.176	0.861
Mental health	3.58 ± 0.85	3.58 ± 0.86	3.52 ± 0.53	−0.093	0.926

***p < 0.001.

Table 3 shows the associations between physical activity, parental psychological control, basic psychological needs, anxiety and mental health. Physical activity, basic psychological needs and anxiety were significantly correlated with mental health (*r* = 0.148, *p* < 0.05; *r* = 0.218, *p* < 0.01; *r* = −0.318, *p* < 0.01). In addition, our study also found that there was a significant negative correlation between parental psychological control and basic psychological needs (*r* = −0. 150, *p* < 0.05), and there was a significant negative correlation between basic psychological needs and anxiety (*r* = −0. 144, *p* < 0.05).

**TABLE 3:** Correlation matrix for physical activity, parental psychological control, basic psychological needs, anxiety, and mental health.

Variables	Physical activity	Parental psychological control	Basic psychological needs	Anxiety	Mental health
Physical activity	1				
Parental psychological control	−0.073	1			
Basic psychological needs	0.103	−0.150*	1		
Anxiety	−0.055	0.086	−0.144*	1	
Mental health	0.148*	−0.015	0.218**	−0.358**	1

**p < 0.01, *p < 0.05.

Table 4 shows the regression coefficients of anxiety mediators. The results showed that basic psychological needs were significantly negatively correlated with anxiety and positively correlated with mental health. Meanwhile, anxiety was negatively correlated with mental health.

**TABLE 4:** Regression coefficients of the mediating of anxiety between basic psychological needs and mental health.

Outcome variables		Goodness-of-fit indices			Regression coefficient and significance	
	Predictors	R	R^2^	*F*	β	*t*
Mental health	Basic psychological needs	0.218	0.048	12.569***	0.218	7.369***
Anxiety	Basic psychological needs	0.144	0.021	5.307**	−0.144	8.533**
Mental health	Basic psychological needs	0.396	0.157	23.280***	0.170	2.903***
	Anxiety				−0.334	−5.694***

***p < 0.001, **p < 0.01

**FIGURE 2:**
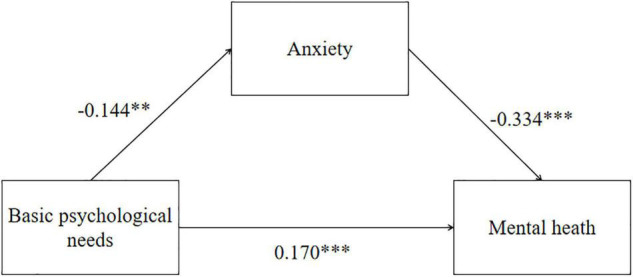
Mediation effect analysis of anxiety between basic psychological needs and mental health; ***p < 0.001, **p < 0.01.

Mediation analysis based on 5,000 bootstrap samples was conducted to estimate the indirect effects of basic psychological needs on mental health mediated by anxiety. Table 5 illustrates the results of the mediation analysis. The direct effect of basic psychological needs on mental health was significant (95% CI: 0.095–0.495), and the indirect effect was also significant (95% CI: 0.017–0.086). The indirect effect of basic psychological needs on mental health via the mediation of anxiety was 0.378 (95% CI: 0.168–0.588) (Table 5 and Figure 2). The results revealed that there is mediating effect of the anxiety between basic psychological needs and mental health.

**TABLE 5:** Mediating effects of anxiety between basic psychological needs and mental health by process.

Effect types	Path	95% CI	Effect
Direct effect	Basic psychological needs→Mental health	0.095-0.495	0.295
Indirect effect	Basic psychological needs→Anxiety→Mental health	0.017-0.086	0.083
Total effect	—	0.168-0.588	0.378

*Demographic variables as covariance.*

## Discussion

The current investigation examined the cross-sectional links between physical activity, basic psychological needs, anxiety, and mental health in Chinese engineering college students during the COVID-19 pandemic and investigated the mediation effect of anxiety between basic psychological needs and mental health. In the current study, we found that physical activity, basic psychological need was positively associated with mental health, while anxiety was negatively correlated to mental health. Besides, anxiety played a mediation effect in the relationship between basic psychological needs and mental health. The present study suggests that gender differences exist in overweight among Chinese engineering college students. Similarly, Shan et al. (2019) investigated 536 undergraduate students in Shijiazhuang, China, and found that there was a significant gender difference in obesity or overweight among male and female college students. Males were more likely to gain weight than females. The reason may be that men eat more sweets, engage in more sedentary behaviors (Zhang et al., 2020) such as playing video games and have fewer opportunities for physical activity during the pandemic period.

The findings of the present study reveal a significant positive correlation between physical activity and mental health. The mental health benefits of regular physical activity and exercise are undisputed in the literature, and there is a wealth of evidence to support participation in physical activity to enhance mental health and reduce some of the negative effects on mental health such as physical dissatisfaction, depression, and perceived stress (Knapen et al., 2015; Alhaqbani et al., 2020; Herbert et al., 2020). Engineering college students are confronted by considerable stress concerning academic demands, adjusting to life away from home and occupational future, etc. (Chen, 2015). Timely physical activities can help them to get rid of their negative emotions, which is conducive to their mental health development. Al Dhaheri et al. (2021) recommend physical activity and exercise as a therapeutic approach to combat the negative mental consequences of isolation during COVID-19. Our study revealed a significantly positively correlation between basic psychological needs and mental health of engineering college students. Due to the COVID-19 pandemic, some global higher education institutions turned to emergency distance learning or other physical and social distancing measures in early 2020, which could compromise the university students’ basic psychological needs (Pelikan et al., 2021). Our results are consistent with prior literature, which have confirmed that satisfying basic psychological needs is beneficial to the healthy mental growth of individuals, such as life satisfaction or enthusiasm (Barber et al., 2005; Howell et al., 2011; Aldrup et al., 2017; Zhou et al., 2017; Ren and Jiang, 2021). Due to their unique disciplinary characteristics, engineering students spend most of their time in the laboratory (Jensen and Cross, 2021), and they may need to satisfy their basic psychological needs (such as relationship needs). Some measures taken by the government or schools to meet their basic psychological needs may be beneficial to their mental health development. Schools can be encouraged to create conditions (such as psychological counseling or physical activity) for engineering students to engage in activities that cultivate their autonomy and ability, and develop more interpersonal skills in the activities, so as to satisfy basic psychological needs at the source.

Our study confirmed that anxiety is negatively correlated with the mental health of engineering college students, which is consistent with prior studies. Studies have shown that both general health anxiety and specific anxiety can affect mental health (Kashiwazaki et al., 2020). Anxiety is a common mental disorder, which can cause physical and mental problems in patients, including sleep disorder, attention deficit, and even suicide intention (Michelsen et al., 2017). Consistent with the findings of Nurunnabi et al. (2020), our study found that female college students experienced more severe anxiety symptoms than male college students during COVID-19 (Hou et al., 2020). This might have occurred because female college students are less adaptable to the epidemic and bear higher pressure, so the severity of anxiety is higher. Engineering college students reported that their lack of sleep, intense competition, lifestyle changes, and other important stressors throughout their undergraduate education could further raise their stress and anxiety levels, which could be detrimental to their mental health (Ross et al., 1999). At present, there are researches about parental psychological control on children and adolescents (Shek et al., 2018; Shek and Dou, 2020), but few studies discuss whether parental psychological control will have different degrees of influence on college students. One primary finding of our study is that there was no correlation between parental psychological control and the mental health of engineering college students, which may be because most engineering college students live on campus and are independently busy with their studies, thinking and life, so parental psychological control has little influence on them.

The current COVID-19 pandemic has brought about an economic crisis that may see paid employment increasingly disappear and liquidation of organizations (Adewumi, 2021), which may have an impact on the psychological state of university students. Research suggests that about 45% of students may have symptoms of acute stress, anxiety or depression during the COVID-19 pandemic (Ma et al., 2020). Our results revealed that anxiety plays a mediating role in the relationship between basic psychological needs and mental health of engineering college students. Our findings support previous researches that foundational psychological needs are negatively correlated with anxiety and positively correlated with mental health (Deci et al., 2001; Van den Broeck et al., 2016). According to the two-process model of needs presented by Sheldon et al. under the framework of self-determination theory (Sheldon, 2011), if an individual’s basic psychological needs are not satisfied, the individual will not be able to conduct normal self-regulation. Given this, if the basic psychological needs of engineering college students cannot be satisfied, they may appear maladaptive state, and even feel anxious (Bartholomew et al., 2011; Quested et al., 2011). Engineering college students are twice as likely to experience psychological problems such as emotion and anxiety as ordinary college students (Danowitz and Beddoes, 2018). Anxiety is negatively correlated with mental health, and the development of anxiety may lead to a low level of mental health (Zhou et al., 2017), which is not conducive to their physical and mental development. In other words, the satisfaction of basic psychological needs can further affect mental health by affecting anxiety.

Theoretically, such mediation models support self-determination theory, which suggests that Once the basic psychological needs of individuals are not satisfied, they will fall into a state of maladaptation. Practically, this study is of great significance for guiding engineering college students to reduce anxiety and promote their mental health development. Despite these findings, the current research is not without limitations. The most important limitation of these analyses is the use of cross-sectional samples; some universities closed completely and switched to online learning due to the epidemic control measures and a few allowed engineering college students to practice in LABS or take physical education classes outdoors, which added to the difficulty of collecting samples; real mediation requires longitudinal data to determine time priorities. Second, the sample size of the study is relatively small. Our study found only gender differences in physical activity among engineering college students, and the results require further studies in a large sample of them. Finally, self-reported data has a possibility for reporting bias.

## Conclusion

To our knowledge, this is the first study to examine the relationships between physical activity, parental psychological control, basic psychological needs, anxiety, and mental health in Chinese engineering college students during the COVID-19 pandemic. In conclusion, there is a significant correlation between physical activity, basic psychological needs, anxiety, and mental health in Chinese engineering college students during the COVID-19 pandemic, meanwhile, anxiety plays a mediation role in the relationship between basic psychological needs and mental health. Government or educators can guide and help students’ basic psychological needs met, in the true sense as to encourage and create conditions for college students to cultivate their autonomy and capacity and meet the need of social activities (design specialized online courses and activities, such as network counseling courses, home-based exercise guidance), on the source satisfy basic psychological needs, thus reducing the anxiety level and promote their mental health. These findings suggest potential mechanisms through which the satisfaction of basic psychological needs can improve Chinese engineering colleges students’ mental health, and targeted interventions can be developed to improve their mental health. School administrators should also pay more attention to students whose family homes have been severely affected by the outbreak. The current study expands the literature on physical activity, basic psychological needs and mental health during the COVID-19 pandemic and is helpful to guide engineering university students to reduce anxiety and promote the development of their mental health. Future studies in college students who are not majoring in engineering or adolescents are awaited.

## Data Availability Statement

The raw data supporting the conclusions of this article will be made available by the authors, without undue reservation.

## Ethics Statement

The studies involving human participants were reviewed and approved by the Ethics Committee of Shandong University (No. 20190912). The patients/participants provided their written informed consent to participate in this study. Written informed consent was obtained from the individual(s) for the publication of any potentially identifiable images or data included in this article.

## Author Contributions

XZ and ZL designed and implemented this study, collected, sorted out, and analyzed the data, and wrote the introduction and methods section. All authors participated in the statistical analysis phase of the project, wrote the results part of the manuscript, provided detailed feedback on other parts of the manuscript, and approved the final clause.

## Conflict of Interest

The authors declare that the research was conducted in the absence of any commercial or financial relationships that could be construed as a potential conflict of interest.

## Publisher’s Note

All claims expressed in this article are solely those of the authors and do not necessarily represent those of their affiliated organizations, or those of the publisher, the editors and the reviewers. Any product that may be evaluated in this article, or claim that may be made by its manufacturer, is not guaranteed or endorsed by the publisher.

## Footnotes

1
https://www.wjx.cn/
